# Benefits of combined exercise training on arterial stiffness and blood pressure in spontaneously hypertensive rats treated or not with dexamethasone

**DOI:** 10.3389/fphys.2022.916179

**Published:** 2022-08-15

**Authors:** Lidieli P. Tardelli, Francine Duchatsch, Naiara A. Herrera, Thalles Fernando R. Ruiz, Luana U. Pagan, Carlos A. Vicentini, Katashi Okoshi, Sandra L. Amaral

**Affiliations:** ^1^ Joint Graduate Program in Physiological Sciences, PIPGCF UFSCar/UNESP, São Carlos, SP, Brazil; ^2^ Department of Physical Education, São Paulo State University (UNESP), School of Sciences, Bauru, SP, Brazil; ^3^ Joint Graduate Program in Animal Biology, São Paulo State University (UNESP), São José do Rio Preto, SP, Brazil; ^4^ Department of Internal Medicine, São Paulo State University (UNESP), Botucatu Medical School, Botucatu, SP, Brazil; ^5^ Department of Biological Sciences, São Paulo State University (UNESP), School of Sciences, Bauru, SP, Brazil

**Keywords:** pulse wave velocity, aerobic training, resistance training, collagen deposition, hypertension, arteries

## Abstract

Dexamethasone (DEX)-induced arterial stiffness is an important side-effect, associated with hypertension and future cardiovascular events, which can be counteracted by exercise training. The aim of this study was to evaluate the mechanisms induced by combined training to attenuate arterial stiffness and hypertension in spontaneously hypertensive rats treated or not with dexamethasone. Spontaneously hypertensive rats (SHR) underwent combined training for 74 days and were treated with dexamethasone (50 µg/kg *s. c*.) or saline solution during the last 14 days. Wistar rats were used as controls. Echocardiographic parameters, blood pressure (BP) and pulse wave velocity (PWV), as well as histological analyses of the heart and aorta, carotid and femoral arteries were performed. At the beginning, SHR had higher BP and PWV compared with Wistar rats. After 60 days, while BP increased in sedentary SHR, combined exercise training decreased BP and PWV. After 74d, the higher BP and PWV of sedentary SHR was accompanied by autonomic imbalance to the heart, cardiac remodeling, and higher arterial collagen deposition. DEX treatment did not change these parameters. On the other hand, trained SHR had reduced BP and PWV, which was associated with better autonomic balance to the heart, reduced myocardial collagen deposition, as well as lower arterial collagen deposition. The results of this study suggest that combined training, through the reduction of aortic collagen deposition, is an important strategy to reduce arterial stiffness in spontaneously hypertensive rats, and these lower responses were maintained regardless of dexamethasone treatment.

## 1 Introduction

Arterial stiffness, indexed as pulse wave velocity (PWV), has been recognized as an integrated predictive marker of age-associated organ damage and as a predictor of future cardiovascular events, as well as all-cause mortality (Scuteri et al., 2020). Studies have shown that PWV is a noninvasive technique that reflects the long-term effect of the established risk factors on the arterial wall, like diabetes, atherosclerosis, metabolic syndrome and hypertension (Lambrinoudaki et al., 2018; Lithovius et al., 2018; Putarek et al., 2018). Although it is unclear whether arterial stiffness is dependent or independently associated with hypertension (Lindesay et al., 2018), vascular alterations are commonly observed in primary or secondary types of hypertension (Scandale et al., 2020; Tardelli et al., 2021). These changes are in fact related to an imbalance between collagen and elastin (components of the extracellular matrix) and stiffness of the vascular smooth muscle cells, which determines the remodeling of the vessels (Jordao et al., 2011; Lacolley et al., 2017).

Our group and others have demonstrated that hypertension induced by chronic treatment with dexamethasone (DEX), a synthetic glucocorticoid, is commonly accompanied by autonomic imbalance to the heart, which is characterized by an increase in low-frequency (LF) and decrease in high-frequency (HF) bands, higher sympathetic drive to the vessels, reduced baroreflex activity, skeletal muscle microcirculation rarefaction and arterial stiffness in wistar normotensive rats (Herrera et al., 2016; Constantino et al., 2017; de Salvi Guimaraes et al., 2017; Herrera et al., 2017; Jesus et al., 2020; Tardelli et al., 2021). Recently, we confirmed these responses in normotensive rats and revealed that DEX (at lower doses) did not exacerbate BP or arterial stiffness in SHR, as it does in normotensive rats (Duchatsch et al., 2021; Tardelli et al., 2021). In fact, DEX improved cardiac remodeling in SHR (Duchatsch et al., 2021). In addition, our group showed that aerobic exercise training decreases BP and improves cardiac function due to myocardial capillary angiogenesis and reduction of myocardial collagen deposition area in SHR treated with DEX (Duchatsch et al., 2021).

Regular exercise training is an effective non-pharmacological therapy to control hypertension (Pescatello et al., 2015; Whelton et al., 2018; Williams et al., 20182018; Unger et al., 2020; Barroso et al., 2021) and is able to attenuate pathological cardiac remodeling (Cattadori et al., 2018) and to improve arterial stiffness (Lopes et al., 2021) in hypertensive humans. Combined exercise training, which consists of aerobic and resistance exercise on alternate days, has been associated with better responses in controlling cardiovascular risk factors for cardiovascular diseases (Figueroa et al., 2011; Ho et al., 2012; Schroeder et al., 2019; Dias et al., 2020a; Aminuddin et al., 2021). However, the impact of combined exercise training on arterial stiffness and cardiac remodeling in SHR are still unclear. Furthermore, nothing was known about the effects of a combined training on arterial stiffness, cardiac remodeling, and hemodynamics in SHR treated with DEX. Thus, the hypothesis of this study was that combined training could decrease BP and PWV through better autonomic balance for the heart, cardiac remodeling, and lower aortic, carotid, and femoral collagen deposition in SHR. In addition, trained SHR would maintain these lower BP and PWV regardless of DEX treatment. The aim of this study was to evaluate the mechanisms induced by combined training to attenuate arterial stiffness and hypertension in spontaneously hypertensive rats treated or not with dexamethasone.

## 2 Methods

### 2.1 Animal care

Fifty-one male SHR (12 weeks) were obtained from the Institute of Biomedical Sciences of the University of São Paulo (São Paulo, SP). Thirteen male Wistar rats (12 weeks) were obtained from the animal breeding facility at São Paulo State University (Botucatu, SP), which were used just to ensure that SHR were indeed hypertensive. All animals were housed in collective cages (5 each) at the animal facility from the School of Sciences, São Paulo State University (UNESP, Bauru, SP), in temperature-controlled room (22°C), with 12:12 h light: dark cycle and free access to water and food. All surgical procedures and experimental protocols were approved by the Ethical Committee for Use of Animals (CEUA, # 775/2017) at the São Paulo State University (UNESP, Bauru, SP) and are in accordance with the Brazilian Ethical Principles in Animal Research.

### 2.2 Groups and pharmacological treatment

After an adaptation period on a treadmill and vertical ladder, all rats were submitted to the maximal exercise capacity test (Tmax) and maximal voluntary carrying capacity (MVCC) test (see below for details). After that, rats were randomly allocated into four groups, which underwent 74 days of the experimental protocol: 1) SHR sedentary control (SC), sedentary during 8 weeks and received daily saline injections (*s.c.*) during the following 14 days (*n* = 11); 2) SHR sedentary DEX (SD), sedentary during 8 weeks and received daily DEX injections (Decadron, *s. c.*) during the following 14 days (*n* = 12); 3) SHR trained control (TC), 8 weeks of combined training (CT) and received daily saline injections (*s.c.*) during the following 14 days (*n* = 15) and 4) SHR trained DEX (TD), 8 weeks of combined training and received daily DEX injections during the following 14 days (*n* = 13). An extra group of Wistar rats (*n* = 13) was used just for baseline control and was not treated with dexamethasone or subjected to training.

**FIGURE 1:**
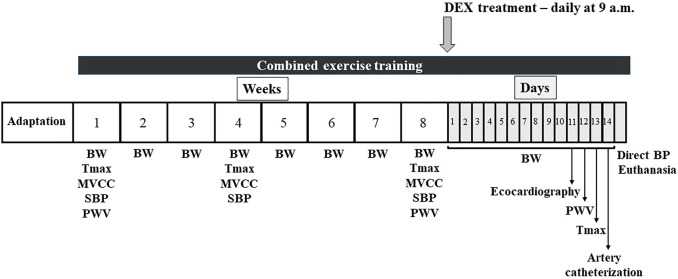
Illustration of the experimental design for 74 days. Adaptation period (5–10 days), body weight (BW), aerobic maximal capacity test (Tmax), maximal carrying capacity test (MVCC), systolic blood pressure (plethysmography) and pulse wave velocity (PWV) were measured throughout the experimental protocol. Combined training was performed during all 74 days. Dexamethasone (DEX) treatment during the last 14 days. At the end of the experimental protocol, echocardiographic and hemodynamic analyses were performed before euthanasia.

Treatment with dexamethasone (Decadron^®^, 50 μg/kg of body weight, *s. c*. Aché Laboratories, Guarulhos, SP, Brazil) was daily performed in SHR rats at 9 a.m. for 14 days (once per day) and control SHR received saline at the same volume as DEX. This DEX dosage was elected based on our previous results, because it showed less undesirable side effects in Wistar rats and did not increase BP in SHR (Tardelli et al., 2021). Body weight was weekly measured during training period (8 weeks) and daily measured during DEX treatment period, as shown in Figure 1, which illustrates the experimental design.

### 2.3 Combined training

Aerobic training. All animals were adapted to walk and run for 10 days on a motorized treadmill (Inbramed, Millenium, Brazil) and them performed the maximal test (Tmax), which evaluated the individual physical capacity as previously described (Duchatsch et al., 2021). Aerobic training was performed on a treadmill at low-to-moderate intensity (40–60% maximal speed running of physical capacity) for 1 h per day (Duchatsch et al., 2021), in alternate days with resistance training.

Resistance training. All rats were also adapted to the vertical ladder (110 cm, 80° incline) as published (Krug et al., 2016) and performed the maximal voluntary carrying capacity (MVCC) test, as previously described (Krug et al., 2016). Resistance training was performed on a vertical ladder at low-to-moderate intensity (40–60% of maximal voluntary carrying capacity) (Macedo et al., 2014), in alternate days with the aerobic training.

Combined training consisted of a combination of aerobic and resistance training, in alternate days, as previously described (Dias et al., 2020b). As shown in Figure 1, this training was performed during 74 days (60 days prior to DEX treatment and 14 days concomitant with DEX training). Important to note that sedentary rats were adapted to the treadmill and ladder to maintain their ability to run and climb every 2 weeks, and were subjected to the maximal tests as the trained groups.

### 2.4 Echocardiographic evaluation

All rats were prepared for transthoracic echocardiography, under anesthesia, as previously described (Pagan et al., 2015; Tardelli et al., 2021), on the 11th day of the DEX treatment (Figure 1). After trichotomy of the anterior region of the thorax, the rats were positioned in the left lateral decubitus position, and the evaluation of the mitral and aortic transvalvular flow was performed using a multifrequency transducer operating at 5.0 MHz, connected to the General Electric Medical Systems equipment, model Vivid S6 (Tirat Carmel, Israel). The evaluated variables were: LV diastolic and systolic dimensions (LVDD and LVSD, respectively), LV mass (LVM), relative wall thickness (RWT), posterior wall shortening velocity (PWSV), myocardial performance index (TEI index), LV ejection fraction (LVEF), E/A ratio between early (E)-to-late(A) diastolic mitral inflow (E/A) and isovolumetric relaxation time (IVRT).

### 2.5 Pulse wave velocity analysis (PWV)

At the beginning and on the 12th day of the experimental protocol (Figure 1), PWV assessments were performed following the procedures already published by our group (Fabricio et al., 2020; Miotto et al., 2021a; Tardelli et al., 2021). In summary, the two pOpet^®^ probes (Axelife SAS, Saint Nicolas de Redon, France) were positioned on the right forelimb and hindlimb of the anesthetized rats. In a quiet room, transit time signal (TT, s), between arteries (carotid-femoral path), was recorded during 10 s by pOpet 1.0 software and PWV was calculated, following the formula: PWV (m/s) = D (m)/TT (s), where D was the distance between probes. Average of 10 measurements was calculated.

### 2.6 Hemodynamics analysis

#### 2.6.1 Tail-cuff plethysmography

All animals were adapted for 5 days to the cylindrical acrylic tube that kept the rats at rest and, during the 8-weeks period of combined training, systolic blood pressure assessments were performed at the beginning, after 30 and 60 days, as stated in Figure 1, following the procedures already published (Miotto et al., 2021a; Miotto et al., 2021b). A cuff was positioned around the tail of the animals and systolic blood pressure (SBP) was recorded by tail-cuff plethysmography system (PanLab LE5001, Barcelona, Spain). In summary, after detection of the pulse, the cuff was inflated to a BP of 300 mmHg. Then, during the process of deflation, the first detected pulse was considered as SBP. Basal SBP was considered from the average of five measurements.

#### 2.6.2 Direct measurements

As shown in Figure 1, on the 14th day of the treatment with DEX, all rats were anesthetized and the carotid artery was catheterized for direct BP measurements following the procedures previously described by our laboratory (Herrera et al., 2016; Constantino et al., 2017; Herrera et al., 2017; Duchatsch et al., 2018; Jesus et al., 2020; Miotto et al., 2021a; Miotto et al., 2021b; Duchatsch et al., 2021; Tardelli et al., 2021). On the next day, BP was recorded in conscious freely moving rats using a Software LabChart Pro 7.1 (ADInstruments, NSW, Australia). From pulsatile measurement of BP, systolic (SBP), diatolic (DBP) and mean (MBP) was calculated. Also, heart rate (HR) was depicted from pulsatile BP.

Variability of HR within frequency domain was calculated using a specific software (CardioSeries V2.7) for spectral analysis, as previously described (Herrera et al., 2016; Duchatsch et al., 2018; Miotto et al., 2021b). In summary, both low-frequency (LF) and high-frequency (HF) bands were integrated from the spectra and cardiac sympathovagal balance was expressed as LF/HF ratio.

### 2.7 Morphological/morphometric evaluations

#### 2.7.1 General information

At the end of the experimental protocol, rats were deeply anesthetized and submitted to a transcardiac perfusion with 4% paraformaldehyde, following the procedures previously described by our laboratory (Fabricio et al., 2020). In detail, myocardium and vessels (thoracic aorta, carotid, and femoral arteries) were removed, fixed in a solution of 4% buffered paraformaldehyde and stored in the refrigerator at 8°C for 24 h.

Subsequently, the tissues were washed for five consecutive days in ethanol (70%) to remove excess paraformaldehyde and then prepared for the Paraplast embedding process (polyisobutylene paraffin mixture, Sigma-Aldrich). The Paraplast^®^ embedding process begins with the tissues being dehydrated in ethanol at concentrations of 95% (2 steps) and 100% (3 steps) for a period of 30 min each step. Soon after, the diaphanization process begins to clean and clarify the tissues with xylene (3 steps of 30 min each). Then, the process of inclusion in paraplast is carried out in the oven (57°-60°C), in three steps of 30 min each and, from there, the samples are blocked and left at room temperature for 24 h.

Serial transverse sections of each tissue were performed using a manual microtome (Thermo/Microm HM 325 Rotary Microtome, Artisan Technology Group, United States) with disposable blades. For tissues, three to six sections were mounted on glass slides.

All images were captured using a camera coupled to the microscope (Leica Microsystems, GmbH, Wetzlar, Germany), at 25–400X magnification, depending on the tissues, as previously published (Tardelli et al., 2021). For all tissues, three photos were taken from each section, totaling 9 to 18 images per tissue of each animal.

All off-line analyses, using ImageJ software, were blindly performed to avoid any misinterpretation by the evaluator.

#### 2.7.2 Myocardium

Myocardial sections of 7 µm thick were stained with Picrosirius Red (Sigma Aldrich, United States) for collagen analysis. The staining steps of the sections were performed as follows: xylene (5 min), xylene (5 min), 100% alcohol (3 min), 90% alcohol (3 min), 80% alcohol (3 min), 70% alcohol (3 min) and washed in running distilled water (10 min). Then, the slides were immersed in Picrosirius red solution for 1 h. To remove the excess of Picrosirius Red, slides were washed in two steps of acidified water (0.5%) for 5 min, followed by three steps of 100% alcohol (3 min), 100% xylene-alcohol (5 min) and two steps of xylene (5 min).

**FIGURE 2:**
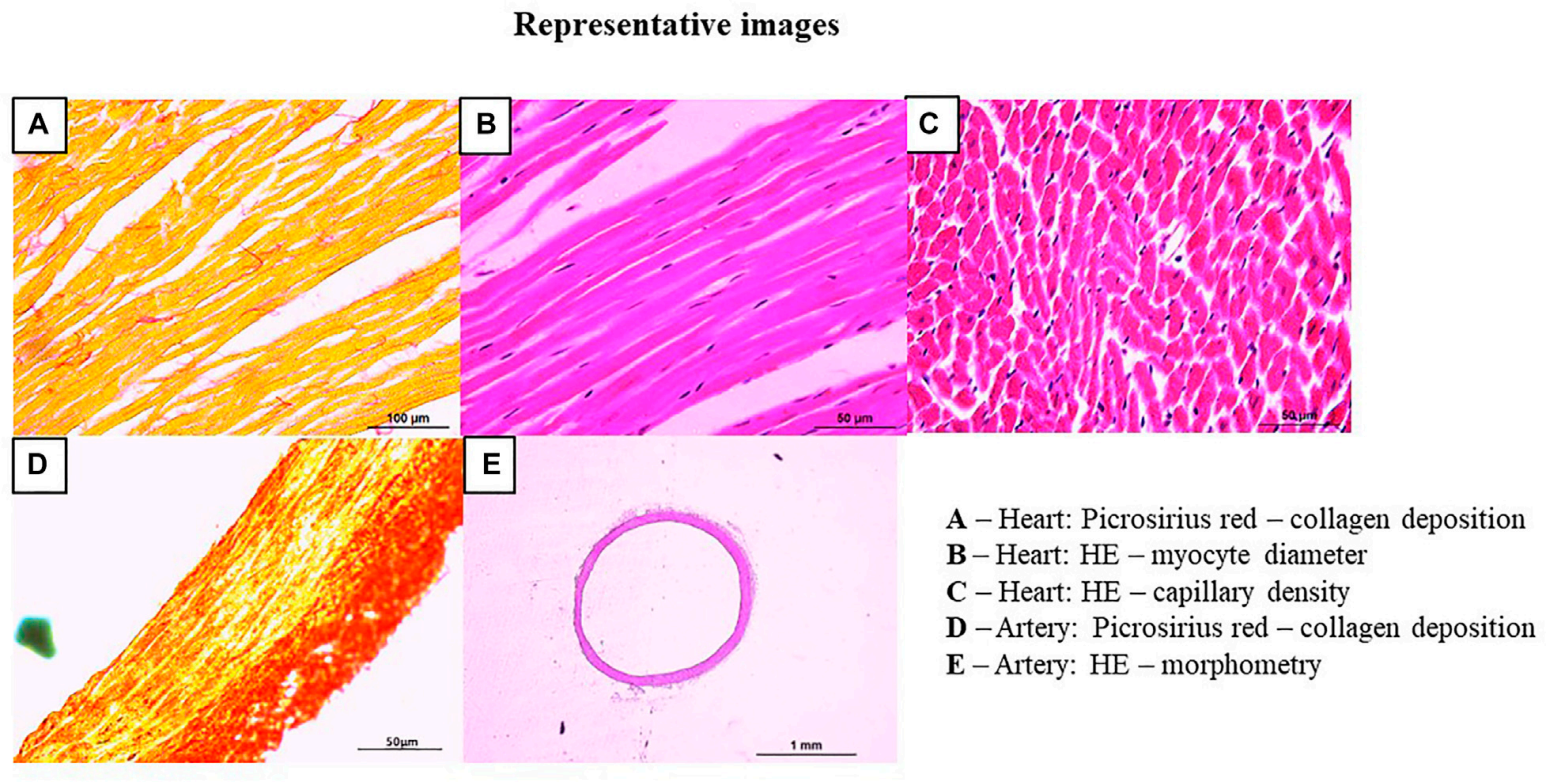
Representative images of histological analyses of the heart and arteries. Panel **(A)** myocardial collagen deposition: myocardial longitudinal sections of 7 µm thick were stained with Picrosirius Red and documented at ×400 magnification: myocytes can be identified in yellow and the fibers of collagen in red (bar-100 µm); Panel **(B)** myocyte diameter: myocardial longitudinal section of 5 µm thick were stained with hematoxylin and eosin staining and documented at ×200 magnification (bar-50 µm); Panel **(C)** myocardial capillary density: myocardial transversal section of 5 µm thick were stained with hematoxylin and eosin staining and documented at ×200 magnification (bar-50 µm); Panel **(D)** arterial collagen deposition: arterial transverse sections of 5 µm thick were stained with Picrosirius Red and documented at ×200 magnification: smooth muscle (in yellow) and collagen fibers (in red) (bar-50 µm); Panel **(E)** vessel morphometry: arterial section of 10 µm thick were stained hematoxylin and eosin staining and documented at ×25 magnification (bar-1mm).

All myocardial sections for Picrosirius Red staining were documented at 400X magnification. A representative image of myocardial section, stained with Picrosirius Red, is illustrated in Figure 2A. In this image, myocytes can be identified in yellow and the fibers of collagen in red. The quantification of the myocardial collagen area and the percentage (%) of the collagen area were automatically calculated by the ImageJ software, based on the detection of red staining in a given area, as published (Miotto et al., 2021b; Duchatsch et al., 2021; Tardelli et al., 2021).

Myocardial sections of 5 µm thick were stained with hematoxylin and eosin staining (H&E, Easy Path, SP) for analysis of myocyte diameter (longitudinal section) and capillary density (transversal section), as published (Herrera et al., 2016; Miotto et al., 2021b; Duchatsch et al., 2021; Tardelli et al., 2021). The steps of HE staining were performed as follows: xylene (5 min), xylene (5 min), 100% alcohol (3 min), 90% alcohol (3 min), 80% alcohol (3 min), 70% alcohol (3 min) and washed in running distilled water (10 min). Then, the slides were immersed in hematoxylin (10 min), washed in running distillated water (10 min), immersed in eosin (7 min) and washed again in running distillated water (3x). Finally, the slides were washed in three steps of 100% alcohol (3 min), 100% xylene-alcohol (5 min) and two steps of xylene (5 min). For these analyses, sections were documented at 200X magnification. The myocyte diameter, illustrated in Figure 2B, was identified in longitudinal sections, after recognizing the membranes (between one myocyte and another) and the nucleus. From a straight line drawn between the membranes, passing through the nucleus, the analysis of the myocyte diameter (µm) was obtained, as previously demonstrated (Rossoni et al., 2011).

Capillary density, illustrated in Figure 2C, was analyzed in H&E-stained tissue determined by the number of capillaries in the transverse section (0.055488 µm^2^). Then, the area of the image was normalized by mm^2^ and the capillary density was expressed as number of capillaries/mm^2^ (Rossoni et al., 2011).

#### 2.7.3 Aorta, carotid and femoral arteries

Sections of 5 µm thick were performed by disposable blades, placed on glass slides, and stained with Picrosirius Red staining (Sigma Aldrich, United States) for collagen analysis, which was identified by smooth muscle (in yellow) and collagen fibers (in red), as shown in Figure 2D. The steps of staining the arteries with Picrosirius Red were the same used for the myocardium. All sections were documented at 200X magnification. The quantification of the collagen area and the percentage (%) of the area of collagen were automatically calculated by the ImageJ software, based on the detection of red staining in a given area, as published (Tardelli et al., 2021).

Sections of 10 µm thick were performed by disposable blades, placed on glass slides and later be stained with H&E for morphometric analyses. The HE staining steps were the same used for the myocardium. All sections were documented at 25X magnification (Figure 2E). The morphometric analysis of the arteries included: outer diameter (OD), inner diameter (ID). From these values, the wall thickness was calculated [(OD-ID)/2], µm) and the wall/lumen ratio (W/L), as previously published (Fabricio et al., 2020; Miotto et al., 2021b; Tardelli et al., 2021).

### 2.8 Statistical analysis

Results are presented as mean ± standard error of the mean (SEM). Two-way analysis of variance (ANOVA) was used to compare four SHR groups. Two-way ANOVA RM was used for analyses along the training and treatment period. Tukey post-hoc test was used when necessary. Student’s t-test was used to compare two groups. Pearson correlation tests were used to associate two parameters (*p* < 0.05).

## 3 Results

### 3.1 Effects of hypertension

Table 1 shows the comparison of body weight, hemodynamics, cardiac and vascular parameters between SHR and WISTAR rats. As shown, SHR had higher SBP (+110%, *p* < 0.001), DBP (+96%, *p* < 0.001) and MBP (+100%, *p* < 0.001) compared with Wistar rats. This response was accompanied by higher LF (+107%, *p* < 0.01), lower HF (-14%, *p* < 0.003) and higher LF/HF (+100%, *p* < 0.01) compared with WISTAR rats. Similarly, as shown in Table 1, the majority of the cardiac structural parameters were worst in SHR, accompanied by preserved systolic function (PWSV, -9%, *p* < 0.005), but not diastolic function. Although myocyte diameter and myocardial collagen deposition area were not significantly altered by hypertension, the number of the myocardial capillaries was lower in SHR compared with Wistar rats (-25%, *p* < 0.0001). In addition, Table 1 revealed that estimated PWV was higher in SHR (+40%, *p* < 0.0001 v*s*. WISTAR) as well as vessel collagen deposition area in all three analyzed arteries: aorta (+43%, *p* < 0.0008), carotid (+52%, *p* < 0.0004) and femoral (+36%, *p* < 0.01). Results of aortic, carotid and femoral morphometric analyses were similar between SHR and WISTAR groups, as shown in Table 1. A more detailed data can be found at Supplementary Table S4.

**TABLE 1:** Comparison of body weight, hemodynamics, cardiac and vascular parameters between hypertensive and normotensive sedentary animals.

	WISTAR	SHR
**Body Weight**		
BW (g)	437 ± 14	331 ± 6 #
**Hemodynamic Analyses**		
SBP (mmHg)	105 ± 2.8	220 ± 3.8 #
DBP (mmHg)	95 ± 4.7	187 ± 10.1 #
LF	13.5 ± 2.3	28 ± 2.4 #
HF	88.3 ± 1.3	76.1 ± 3.4 #
LF/HF	0.17 ± 0.03	0.34 ± 0.05 #
**Myocardial analyses**		
LVSD (mm)	4.28 ± 0.68	3.49 0.48 #
LVDD (mm)	8.06 ± 0.58	7.28 ± 0.42 #
RWT	0.32 ± 0.02	0.41 ± 0.03 #
PWVS (mm/s)	41.55 ± 3.78	37.89 ± 1.4 #
TEI index	0.513 ± 0.05	0.515 ± 0.05
IVRT (ms)	27.38 ± 5.54	28.36 ± 1.74
Myocyte Diameter (µm)	15.8 ± 0.56	16.8 ± 0.95
Capillary Density (n/mm²)	1352.9 ± 40.6	1016.5 ± 13.1 #
% Collagen Area	6.56 ± 0.14	6.67 ± 0.22
**Arterial stiffness and vessel morphometric analyses**		
PWV (m/s)	3.92 ± 0.05	5.49 ± 0.35 #
Aorta artery	—	—
% Collagen Area	14.6 ± 0.83	20.95 ± 1.16 #
Thickness (µm)	99.2 ± 3.95	103.4 ± 3.1
Thickness/lumen ratio	0.065 ± 0.002	0.068 ± 0.001
**Carotid artery**		
% Collagen Area	13.21 ± 1	20.11 ± 0.67 #
Thickness (µm)	48.74 ± 4.63	49.8 ± 2.1
Thickness/lumen ratio	0.067 ± 0.007	0.079 ± 0.004
**Femoral artery**		
% Collagen Area	15.75 ± 1.82	21.4 ± 0.92 #
Thickness (µm)	47.44 ± 2.65	47.8 ± 4.2
Thickness/lumen ratio	0.09 ± 0.006	0.08 ± 0.008

Systolic blood pressure (SBP), Diastolic blood pressure (DBP), Mean blood pressure (MBP), Spectral analysis: low frequency (LF), high frequency (HF), low frequency divided by high frequency in pulse interval (LF/HF), Left ventricle systolic diameter (LVSD), Left ventricle diastolic diameter (LVDD), Relative wall thickness (RWT), Posterior wall shortening velocity (PWSV), Myocardial performance index (TEI index), Isovolumetric relaxation time (IVRT) and Pulse Wave Velocity (PWV) in sedentary animals: normotensive (Wistar, n=13) and hypertensive (SHR, n=11).; Significance: # *vs* Wistar rats.

### 3.2 Effects of combined training in SHR

Table 2 shows the values of body weight and physical capacity during the protocol of combined training and DEX treatment in all SHR Groups. As shown, all groups presented similar BW, aerobic capacity, and resistance capacity at the beginning of the protocol. At the end of training (week 8) all groups presented higher BW compared with week 0 and trained control group had lower BW compared with sedentary control group. At the end, as expected, trained groups had higher aerobic and resistance capacity, compared with their respective sedentary groups.

**TABLE 2:** Parameters of Body Weight and Physical Capacity during combined training and DEX treatment in all SHR Groups.

	SC	SD	TC	TD
**Body Weight (g)**				
Week 0	294 ± 4.7	301 ± 6.2	282 ± 7.6	297 ± 5.8
Week 8	332 ± 5.2 $	338 ± 6.2 $	320 ± 7.9 $+	331 ± 6.9 $
Final	332 ± 6.3 $	291 ± 6.3 †*	318 ± 7.4 $	284 ± 5.3 $†*
**Aerobic Training (s)**				
Week 0	618 ± 41	617 ± 53	702 ± 15	777 ± 40
Week 8	861 ± 77 $	992 ± 75 $	1435 ± 57 $+	1464 ± 47 $+
Final	906 ± 75 $	1024 ± 78 $	1639 ± 85 $†+	1656 ± 74 $†+
**Resistance Training (g)**				
Week 0	317 ± 9	321 ± 11	328 ± 9	336 ± 9
Week 8	585 ± 21 $	571 ± 17.7 $	750 ± 20 $+	762 ± 25 $+
Final	688 ± 24 $†	647 ± 23 $	869 ± 33 $†+	792 ± 26 $+*

Values of body weight (BW, g) and the results of the aerobic capacity (seconds) and resistance capacity (carried weight, g)in all SHR Groups: Sedentary Control(SC, n = 13), Sedentarytreated with DEX (SD, n=10), Trained Control (TC, n = 11) and Trained treated with DEX (TD, n = 11). Significance: $ *vs* Week 0; † *vs* Week 8; * *vs* control; + *vs* Sedentary; *p* <0.05.

**FIGURE 3:**
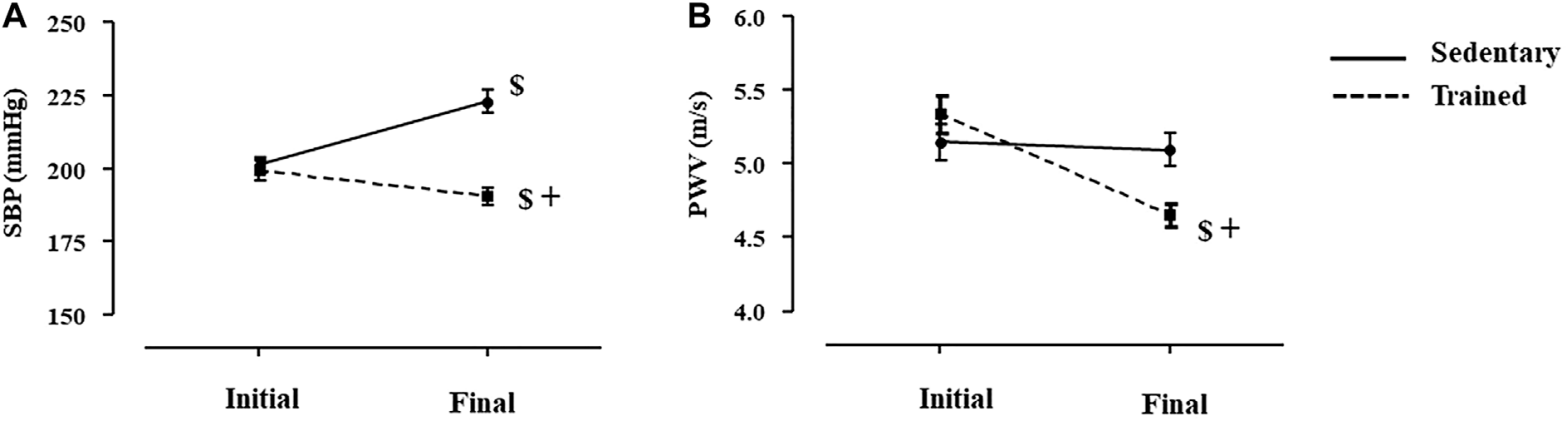
Values of systolic blood pressure **(A**), SBP measured by plethysmography, mmHg) and estimated pulse wave velocity **(B)**, PWV, m/s) at the initial and final moments of the training protocol (8 weeks), before dexamethasone treatment in all SHR groups: Sedentary (n = 23) and Trained (*n* = 28). Significance: $ vs*.* Initial, + vs*.* Sedentary; *p* < 0.05.

As shown in Figure 3, sedentary and trained SHR presented similar values of SBP (3A) and PWV (3B) at the beginning of the protocol. Combined exercise training *per se* decreased SBP (-5%, *p* < 0.02, Panel A) and estimated PWV (-13%, *p* < 0.03, Panel B), when values were compared with the beginning of the training. Note that SBP of sedentary SHR increased 11% during the 8-weeks period and PWV did not change (Figure 3). At the end of the exercise protocol (8-weeks period), both SBP and PWV were lower in trained SHR compared with sedentary SHR.

**FIGURE 4:**
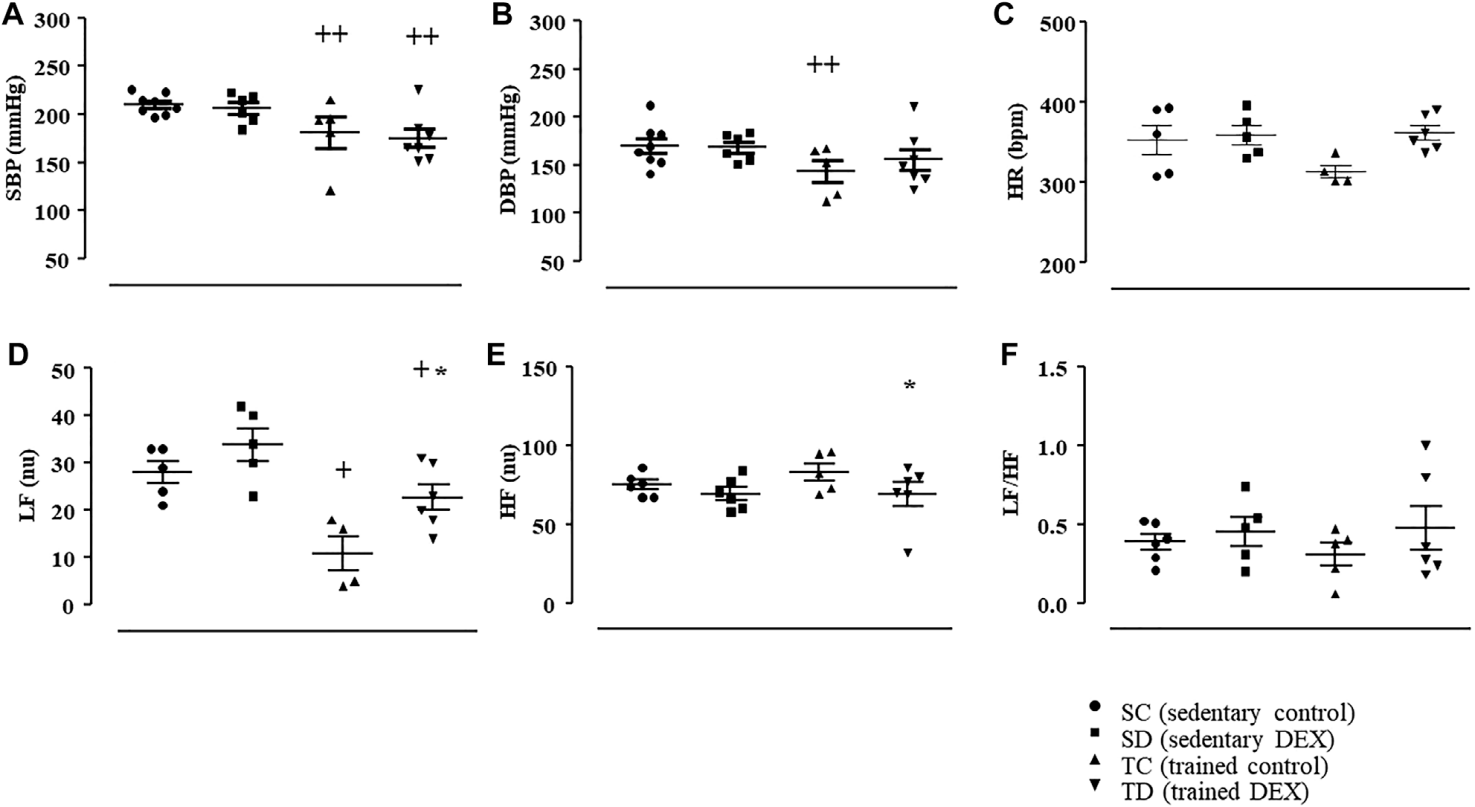
Values of systolic blood pressure (SBP, mmHg, Panel **(A)**; diastolic blood pressure (DBP, mmHg, Panel **(B)**; heart rate (HR, bpm, Panel **(C)**. Analysis of autonomic balance to the heart. Low frequency band (LF, nu, Panel **(D)**; high frequency band (HF, nu, Panel **(E)**; ratio of LF and HF (LF/HF, Panel **(F)** in all SHR groups: Sedentary control (SC, *n* = 6), Sedentary treated with DEX (SD, *n* = 6), Trained control (TC, *n* = 5), Trained treated with DEX (TD, *n* = 7). Significance: * vs*.* Control, + vs*.* Sedentary; *p* < 0.05.

At the end of the experimental protocol, it was possible to observe that trained SHR presented lower values of SBP (-11%, *p* = 0.029, Panel A) and DBP (-23%, *p* = 0.013, Panel B), followed by slightly reduction (but not significant) in HR (-15%, *p* < 0.173, Panel C), compared with sedentary groups, as shown in Figure 4. These responses were possibly induced by the lower LF values found after training in SHR (Figure 4).

In addition, combined exercise training reduced myocardial collagen deposition area (-18%, *p* < 0.001), without affecting other cardiac structures, as observed in Table 3. Myocyte diameter and myocardial capillary density was not affected by combined training in SHR, as shown in Table 3.

**TABLE 3:** Effects on DEX treatment and Combined Training on Myocardial structural parameters, heart function and histological analysis in SHR groups.

	SC	SD	TC	TD
**Structural parameters and LV function**				
LVDD (mm)	7.28 ± 0.12	7.65 ± 0.14	7.09 ± 0.16	7.43 ± 0.12
LVSD (mm)	3.49 ± 0.14	4.04 ± 0.52 *	3.43 ± 0.15	3.7 ± 0.15
LVM (g)	0.73 ± 0.03	0.75 ± 0.03	0.68 ± 0.02	0.67 ± 0.02 +
LVMI (g/kg)	2.18 ± 0.10	2.46 ± 0.09 *	2.13 ± 0.05	2.36 ± 0.07
RWT	0.41 ± 0.01	0.38 ± 0.01 *	0.41 ± 0.01	0.37 ± 0.01 *
PWSV (mm/s)	37.8 ± 1.40	37.9 ± 0.92	36.2 ± 1.03	40.8 ± 0.90 *
Tei index	0.51 ± 0.01	0.50 ± 0.01	0.54 ± 0.01	0.49 ± 0.01
LVEF	0.88 ± 0.01	0.85 ± 0.01	0.88 ± 0.01	0.87 ± 0.01
E/A	1.80 ± 0.17	1.71 ± 0.09	2.02 ± 0.09	1.84 ± 0.04
IVRT (ms)	28.7 ± 0.4	26.6 ± 0.7	30.7 ± 0.5	27.1 ± 0.8 *
**Histological Analysis**				
Myocyte Diameter (µm)	16.8 ± 0.9	15.6 ± 0.5	16.7 ± 0.4	15.5 ± 0.6
Capillary Density (n/mm²)	1016.6 ± 12.2	1117.5 ± 44.5	1042.4 ± 97.7	1076.1 ± 38.6

Left Ventricular Diastolic Diameter (LVDD); Left Ventricle Systolic Diameter (LVSD); Left Ventricular Mass (LVM); Left Ventricular Mass Index (LVMI); Relative Wall Thickness (RWT); Posterior Wall Shortening Velocity(PWSV); Myocardial Performance Index (TEI INDEX); Ejection Fraction (LVEF); E/A ratiobetween early (E)-to-late (A) diastolic mitral inflowand Isovolumetric Relaxation Time(IVRT) in all SHR Groups: Sedentary control(SC, n = 13), Sedentary treated with DEX (SD, n=10), Trained control (TC, n = 11) and Trained treated with DEX (TD, n = 11). Significance: * *vs* Control, + *vs* Sedentary; *p* <0.05.

**FIGURE 5:**
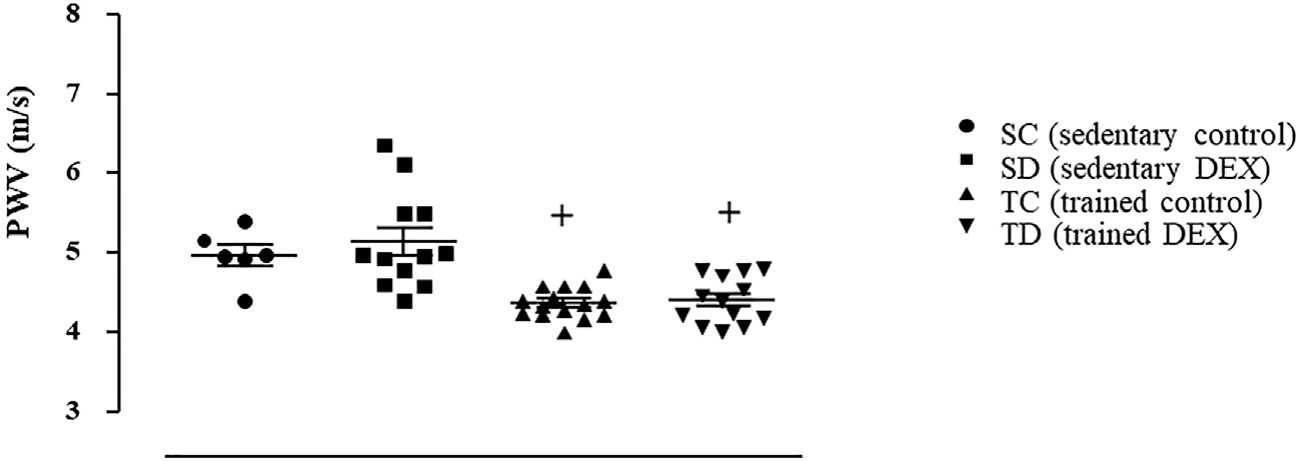
Estimated values of pulse wave velocity (PWV, m/s) at the end of the experimental protocol in all SHR groups. Sedentary control (SC, *n* = 11), Sedentary treated with DEX (SD, *n* = 12), Trained control (TC, *n* = 15), Trained treated with DEX (TD, *n* = 13). Significance: + vs*.* Sedentary; *p* < 0.05.

**FIGURE 6:**
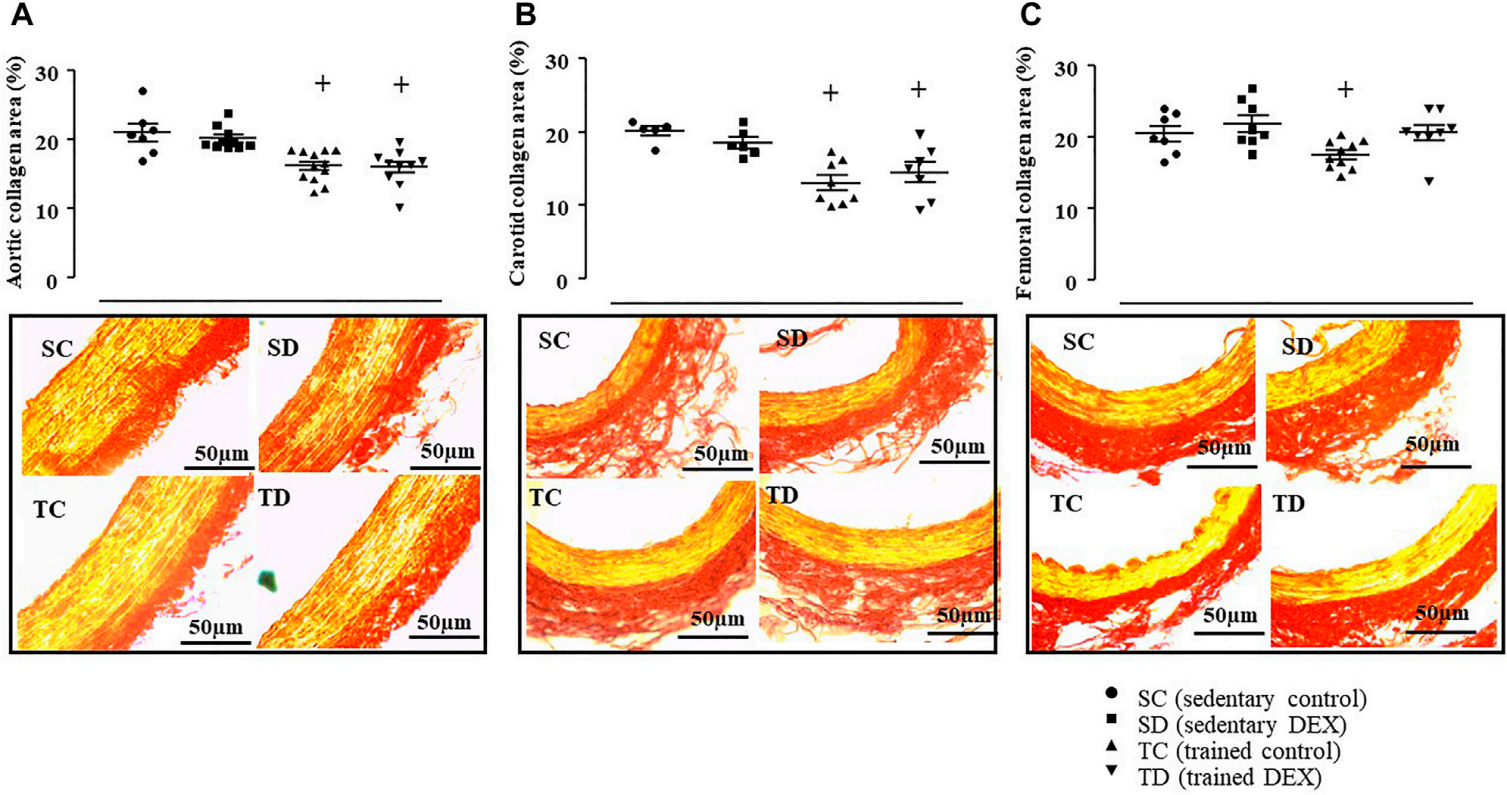
Values of collagen deposition area: aorta artery (%, Panel **(A)**, carotid artery (%, Panel **(B)** and femoral artery (%, Panel **(C)** in all SHR groups: Sedentary control (SC, *n* = 7), Sedentary treated with DEX (SD, *n* = 10), Trained control (TC, *n* = 12), Trained treated with DEX (TD, *n* = 10). Significance: + vs*.* Sedentary; *p* < 0.05. Representative images of collagen deposition area on aorta, carotid, and femoral arteries. Cross-sections (5 μm) taken from one animal of each group stained with Picrosirius-red. Stained in yellow represents the smooth muscle fibers and stained in red represents the collagen fibers. Bar: 50 µm.

Figure 5 illustrates that trained SHR presented lower values of estimated PWV (-20% vs. SC, *p* = 0.001) and this response was accompanied by lower aortic (-23%, Figure 6, Panel A, *p* = 0.001), carotid (-35%, Figure 6, Panel B, *p* < 0.001), femoral (-14%, Figure 6, Panel C, *p* = 0.003) collagen deposition area, compared with sedentary SHR (TC vs*.* SC). On the other side, combined training had no effects on vessel remodeling (Supplementary Table S5).

### 3.3 Effects of DEX treatment in sedentary and trained SHR

Chronic treatment with DEX did not change BP values and HR in sedentary SHR, as shown Figure 4 (panel A–C). In agreement, DEX treatment did not change the values of LF, HF or LF/HF (Figure 4, panel D–F) in sedentary SHR, compared with sedentary control SHR. Interesting, combined training attenuated the increase of LF induced by DEX treatment, since TD group presented lower LF (-21%, *p* < 0.05) compared with SD, which may help to maintain BP lower, even after DEX treatment, as demonstrated in Figure 4.

Table 2 shows that DEX treatment decreased BW in sedentary and trained SHR (final) and did not interfere on the effects of training on physical capacities.

Table 3 shows that DEX treatment slightly changed few cardiac structural parameters in sedentary SHR, like LVSD (+16%, *p* < 0.003), LVMI (+13%, *p* < 0.014) and RWT (-7%, *p* < 0.007) compared with control SHR (SC). Important to note that combined training did not change those cardiac structural parameters that were already altered by DEX, as observed in TD group (Table 3), since LVSD and RWT were similar between SD and TD groups. Only LVM was slightly lower in TD compared with SD (-11%, *p* < 0.05). Table 3 also shows that trained SHR, when treated with DEX, presented higher PWSV (systolic function) and lower IVRT (diastolic function), even though myocardial collagen deposition was similar to those trained SHR (without DEX). Myocyte diameter and myocardial capillary density was not affected by combined training in SHR treated or not with DEX. Even though myocardial collagen deposition area was lower after DEX treatment (-9%, *p* < 0.019 vs*.* SC), these changes were not accompanied by an improvement in cardiac function in SHR.

In addition, as expected, DEX treatment did not increase the estimated PWV (Figure 5) in sedentary SHR. In accordance, it did not change the aortic (Figure 6A), carotid (Figure 6B) or femoral (Figure 6C) collagen deposition areas in sedentary SHR. The bottom panel of Figure 6 illustrates a transverse section of the aorta, carotid or femoral arteries stained with Picrosirius Red. As shown, collagen deposition area (red color) was similar between control and DEX treated SHR. In addition, DEX did not change any of the morphometric parameters of the analyzed vessels (Supplementary Table S5).

Although vessel collagen deposition area (%) was lower in trained SHR compared with sedentary ones, it was not affected by DEX treatment, which helped to maintain estimated PWV at lower values, even after DEX treatment, as shown in Figure 5.

Pearson correlation tests were performed to analyze the possible correlation between collagen deposition and PWV in all SHR groups. The results showed that estimated PWV was positively correlated with aortic collagen deposition area (r = 0.606, *p* < 0.0001), carotid (r = 0.447, *p* = 0.02) and femoral (r = 0.370, *p* < 0.04). In addition, SBP was positively correlated with values of estimated PWV (r = 0.484, *p* < 0.01).

## 4 Discussion

The main findings of this study were that combined training *per se* reduced PWV and BP in SHR and trained rats maintained lower values of PWV and BP, regardless of DEX treatment. In addition, this study revealed that lower PWV in all trained SHR was associated with lower aortic, carotid, and femoral collagen deposition area.

It is already established that SHR is characterized by neural alterations (Bertagnolli et al., 2006; Masson et al., 2015; Duchatsch et al., 2021), accompanied by cardiac remodeling (Pagan et al., 2015; Silva et al., 2017; Li et al., 2019; Duchatsch et al., 2021) and vessel structural and functional alterations (Melo et al., 2003; Amaral and Michelini, 2011), which contribute to increase blood pressure. In the first part of the present study, we confirmed some previous results that SHR had higher BP, accompanied by an autonomic imbalance to the heart. Also, SHR presented lower LVSD, LVDD and higher RWT, followed by myocardial capillary rarefaction. These responses contributed to reduce systolic function, even though myocardial collagen deposition was not significantly higher in SHR, as shown elsewhere (Silva et al., 2017; Miotto et al., 2021b). In addition, this study expanded our previous research (Tardelli et al., 2021) and showed higher deposition of collagen in all three analyzed arteries (aorta, carotid, and femoral arteries), compared with Wistar rats, which was correlated with the higher estimated PWV.

Similarly, our group and others have shown that DEX-induced hypertension is also accompanied by neural alterations (Dodic et al., 1999; Segar et al., 2006; Herrera et al., 2016; Constantino et al., 2017; Herrera et al., 2017; Duchatsch et al., 2018; Macedo et al., 2020; Duchatsch et al., 2021; Tardelli et al., 2021), cardiac remodeling (Dodic et al., 2001; de Salvi Guimaraes et al., 2017; Macedo et al., 2020) and arterial stiffness (Tardelli et al., 2021) and the mechanisms to control hypertension involve an overproduction of reactive oxygen species (Mondo et al., 2006; Ong et al., 2008; Macedo et al., 2020), reduction of nitric oxide (NO) bioavailability (Ong and Whitworth, 2012), autonomic imbalance to the heart (Tardelli et al., 2021) and skeletal muscle capillary rarefaction (Herrera et al., 2016; Herrera et al., 2017; Jesus et al., 2020) in normotensive animals. In addition, DEX treatment may cause either cardiomyocyte hypertrophy (Dodic et al., 2001; de Salvi Guimaraes et al., 2017; Macedo et al., 2020) or benefic myocardial remodeling, especially under pathological situations, like adrenalectomy and hypertension (Narayanan et al., 2004; Xu et al., 2011; Duchatsch et al., 2021). In fact, the different DEX protocols puzzle the interpretations. Furthermore, DEX increases arterial stiffness in Wistar rats, and the mechanisms were still inconclusive. Since vessel stiffness may involve matrix protein abundance on the vessel wall (especially collagen, elastin and fibrillin), reduction of elastic fibers, endothelial dysfunction or alterations on glucose metabolism (Moraes-Teixeira Jde et al., 2010; Araujo et al., 2020; Fabricio et al., 2020; Tardelli et al., 2021), we showed that DEX treatment increases PWV in Wistar rats, mainly through an increase in the deposition of aortic collagen (Tardelli et al., 2021). In the present study, we confirmed some previous results (Tardelli et al., 2021) that DEX did not worsen the autonomic imbalance to the heart, nor did it aggravate myocardial remodeling, cardiac function and arterial stiffness in SHR, which explains the maintenance of BP and arterial stiffness even after DEX treatment.

In the second part of this study, we investigated the effects of combined training on arterial stiffness and hypertension in SHR, treated or not with DEX. Exercise training is a worldwide non-pharmacological strategy to control hypertension, recognized by societies of hypertension (Whelton et al., 2018; Williams et al., 20182018; Sharman et al., 2019; Unger et al., 2020; Barroso et al., 2021). Aerobic exercise is the major component of the training for hypertension and the mechanisms involved are restoration of baroreflex activity, normalization of the autonomic imbalance to the heart (Masson et al., 2016; Ferreira-Junior et al., 2019) reduction of the higher sympathetic drive to the vessels (Li et al., 2019; Miotto et al., 2021b; Duchatsch et al., 2021), skeletal muscle arterioles remodeling (Amaral and Michelini, 2011; Miotto et al., 2021b), and capillary angiogenesis (Amaral and Michelini, 2011; Miotto et al., 2021b) among others. In addition, aerobic training has been shown to prevent and/or reverse arterial stiffness not only in hypertension, but in metabolic syndrome and aging (Moraes-Teixeira Jde et al., 2010; Hasegawa et al., 2018; Guers et al., 2019; Miotto et al., 2021b; Gioscia-Ryan et al., 2021; Lopes et al., 2021). Although some studies have shown some benefits of resistance training *per se* in reducing BP (Perilhao et al., 2020; Feitosa et al., 2021), there is not enough evidence that it reduces ambulatory BP or PWV among the hypertensive population (Li et al., 2015; Hasegawa et al., 2018). Therefore, the recommendation to control hypertension is aerobic training complemented by resistance training (Whelton et al., 2018; Williams et al., 20182018; Sharman et al., 2019; Unger et al., 2020; Barroso et al., 2021), also called combined training. Combined training has been shown to be very effective in improving cardiorespiratory fitness, muscle strength, muscle mass, and inflammatory and oxidative stress parameters (Figueroa et al., 2011; Ho et al., 2012; Schroeder et al., 2019; Dias et al., 2020b), especially during aging (Lee et al., 2015). Recently, Schroeder et al. (Schroeder et al., 2019) compared the effects of aerobic, resistance, and a combination of both aerobic and resistance training on several cardiovascular risk factors, including peripheral and central BP, cardiorespiratory fitness, muscular strength, body composition, blood glucose and lipids and, concluded that, among individuals at an increased risk for cardiovascular disease, 8-weeks of combined training provided more comprehensive cardiovascular benefits compared to time-matched aerobic or resistance training alone.

In fact, there are several clinical studies demonstrating the effectiveness of the combined training for hypertension (Figueroa et al., 2011; Li et al., 2015; Son et al., 2017a; Son et al., 2017b; Pekas et al., 2020), however, the mechanisms induced by combined training to reduce BP and PWV in SHR are not completely understood. The results of this present study showed that combined training *per se* reduced BP in SHR and, the mechanism involved a better autonomic balance to the heart. In accordance, Conti and collaborators and Shimojo and collaborators (Shimojo et al., 2018) showed that combined exercise training improved baroreflex sensitivity and reduced SBP variance, which contributed to reduce BP in a model of metabolic syndrome and menopause. More recently, Dias and collaborators (Dias et al., 2020b) showed that combined exercise training, initiated early in life, mitigated the cardiovascular autonomic dysfunction and BP increase induced by fructose overload in SHR. Since cardiac remodeling is generally associated with hypertension, we analyzed the effects of combined training on myocardial remodeling. Combined training did not improve any of the echocardiographic structural parameters in SHR. Probably, its effects on the structure and function of the heart could be more evident in situations with significant impairment of cardiac function, such as aging, heart failure, metabolic syndrome and menopause, diabetes and others (Conti et al., 2015; Sanches et al., 2018; Shimojo et al., 2018; Dias et al., 2020b). Although combined training in the present study did not increase myocardial capillary density, it reduced myocardial collagen deposition area. Probably it was an initial response, and longer periods of combined training could improve some of the cardiac parameters and function altered in sedentary SHR. Recently, in agreement with these results, we have shown that aerobic training decreased BP in part due to a better autonomic balance to the heart in SHR without any change on cardiac structure (Duchatsch et al., 2021). It seems that the main mechanism to reduce BP in SHR is the skeletal muscle arterioles remodeling (reduction of wall-to-lumen ratio) and capillary angiogenesis, as shown (Melo et al., 2003; Amaral and Michelini, 2011; Williams et al., 20182018; Miotto et al., 2021b). Therefore, the effects of combined training on BP lowering were similar to those seen with aerobic training (Duchatsch et al., 2021).

In addition, combined training reduced estimated PWV in SHR, which was a very positive response, since arterial stiffness is considered an important marker of vessel health, an indicative of subclinical organ damage and predictor for the future cardiovascular events (Scuteri et al., 2020; Shchetynska-Marinova et al., 2021). Clinical measurement of PWV has been suggested to early control the development of arterial stiffness, in both patients with increased cardiovascular risk and the general population (Jannasz et al., 2019), since arterial stiffness may be independent of the BP values (Celik et al., 2017; Guers et al., 2019; Rode et al., 2020). There are some meta-analyzes comparing the effects of different types of exercise (aerobic, resistance, combined and high intensity interval training) on PWV, however they include hypertensive and normotensive individuals (Martinez-Vizcaino et al., 2019), which challenge the interpretation of the results. In agreement with the present results, the clinical relevance of the combined training has been shown in hypertension, aging and other cardiovascular risk factors (Figueroa et al., 2011; Montero et al., 2015; Son et al., 2017b; Pekas et al., 2020), but the underlying mechanisms induced by combined training to decrease arterial stiffness in hypertensive individuals are not completely understood, basically due to inherent challenges of mechanistic studies of large arteries in humans. In addition, most of the human/animal studies investigating the mechanisms of arterial stiffness reduction are performed using aerobic exercise (Moraes-Teixeira Jde et al., 2010; Hasegawa et al., 2018; Kohn et al., 2018; Gioscia-Ryan et al., 2021). Only few studies investigated the mechanisms of combined training on PWV.

This present study showed that combined training reduced aortic, carotid, and femoral collagen deposition area with no changes in vessel remodeling in SHR and, the amount of the collagen on the vessel wall was positively correlated with estimated PWV. Although it is already known that exercise training significantly changes arterioles resistance (Melo et al., 2003; Amaral and Michelini, 2011; Miotto et al., 2021b), in this present study, we performed the studies (morphometric and collagen deposition) on conductance vessels to understand the PWV results. Recently, Otsuki and collaborators (Otsuki et al., 2020) have shown that combined training increased plasma NOx concentration, a marker of nitric oxide production, which was associated with lower PWV, but these authors studied older people and not hypertensive. In agreement, combined training elevated nitrite/nitrate concentration, as well as reduced endotelin-1 in obese and pre-hypertensive girls (Son et al., 2017a). Similarly, Pekas and collaborators (Pekas et al., 2020) had also shown that combined training reduced PWV in older post-menopause woman and this response was associated with an improvement of SBP, low-density lipoprotein, and body fat percentage. Combining the results of the studies mentioned above and the present study, we can suggest that combined training is effective in reducing arterial stiffness through local (collagen deposition in vessels) and systemic changes (plasma concentrations of nitrite/nitrate, endothelin-1 and low-density lipoprotein content).

Some previous results from our group showed that aerobic training, performed earlier and concomitant with DEX treatment in normotensive rats, was effective in mitigating several DEX-induced side-effects, including hypertension (Herrera et al., 2016; Herrera et al., 2017; Jesus et al., 2020). Although we have recently shown that DEX treatment did not further increase BP or PWV in SHR (Tardelli et al., 2021), we investigated the effects of DEX in trained SHR and showed that low dose of DEX, associated or not with aerobic exercise training protected SHR´s heart, but only trained SHR presented lower BP (Duchatsch et al., 2021). In agreement, this present study revealed that DEX did not interfere with the beneficial response of combined training in reducing arterial stiffness and arterial pressure in SHR and, only the association of DEX treatment and combined exercise improved systolic and diastolic function. The results observed with combined training on BP of SHR were similar to those found in our previous study using aerobic training (Duchatsch et al., 2021).

In agreement with our hypothesis, combined training decreased PWV due to lower aortic, carotid, and femoral collagen deposition in SHR. Likewise, BP was lower in trained SHR through better autonomic balance to the heart and likely associated with skeletal muscle arterioles remodeling (data not evaluated in this present study). However, part of the hypothesis was not confirmed, because although combined training decreased myocardial collagen deposition, it did not improve cardiac remodeling induced by hypertension. In addition, the results of the present study confirmed the hypothesis that lower PWV and BP, observed in trained SHR, were maintained regardless of DEX treatment. On the other side, cardiac function was improved in trained SHR only after DEX treatment, probably due to the combination of the DEX anti-inflammatory effects with those induced by combined training (Shimojo et al., 2018; Dias et al., 2020a).

This study has some limitations: first, since combined training reduced BP and PWV, we cannot avoid the possibility that PWV response was dependent of the BP, even though some studies have shown that arterial stiffness reduction may be independent of BP reduction; second, due to the limitation of the technique, PWV was assessed in anesthetized rats. Another limitation of the study was the damping factor due do the catheter-system measurement of BP. Additionally, in this present work, the molecular pathways involved in the vessel collagen deposition process were not evaluated; therefore, other studies *in vivo* and *in vitro* are needed to better clarify our findings.

In conclusion, the results observed in this study revealed, for the first time, that 8-weeks combined training reduced arterial stiffness through less aortic, carotid, and femoral collagen deposition and this reduction, associated with a better autonomic balance to the heart, may contribute to lower BP. This study reinforces the fundamental role of combined training as a non-pharmacological strategy in the control of arterial stiffness and hypertension, independent of DEX treatment.

## Data Availability

The original contributions presented in the study are included in the article/Supplementary Material further inquiries can be directed to the corresponding author.
