# Supplementary material for: Benefits of combined exercise training on arterial stiffness and blood pressure in spontaneously hypertensive rats treated or not with dexamethasone
Source: Front Physiol. 2022 Aug 15;13:916179. doi: 10.3389/fphys.2022.916179 (PMC9420846; doi:10.3389/fphys.2022.916179)
Supplement: Supplementary file 1 [file DataSheet1.pdf]

Table 4S. Comparison of hemodynamics, cardiac and vascular parameters between hypertensive and normotensive sedentary animals

	WISTAR	SHR
Body Weight and Aerobic/Resistance Training		
Final BW (g)	437 ± 14	332 ± 6 #
Final Aerobic capacity (s)	733 ± 36	905 ± 75 #
Final Resistance capacity (g)	489 ± 37	688 ± 24 #
Hemodynamic Analyses		
SBP (mmHg)	105 ± 2.8	220 ± 3.8 #
DBP (mmHg)	95 ± 4.7	187 ± 10.1 #
HR (bpm)	354 ± 21.5	384 ± 17.1
LF (nu)	13.5 ± 2.3	28 ± 2.4 #
HF (nu)	88.3 ± 1.3	76.1 ± 3.4 #
LF/HF	0.17 ± 0.03	0.34 ± 0.05 #
Myocardial analyses		
LVSD (mm)	4.28 ± 0.68	3.49 0.48 #
LVDD (mm)	8.06 ± 0.58	7.28 ± 0.42 #
LVM (g)	0.73 ±0.03	0.73 ± 0.03
LVMI (g/kg)	1.73 ± 0.04	2.18 ± 0.1 #
RWT	0.32 ± 0.02	0.41 ± 0.03 #
PWVS (mm/s)	41.55 ± 3.78	37.89 ± 1.4 #
TEI index	0.513 ± 0.05	0.515 ± 0.05
LVEF	0.84 ± 0.01	0.88 ± 0.01
E/A	1.64 ± 0.1	1.80 ± 0.1
IVRT (ms)	27.38 ± 5.54	28.36 ± 1.74
Myocyte Diameter (µm)	15.8 ± 0.56	16.8 ± 0.95
Capillary Density (n/mm²)	1352.9 ± 40.6	1016.5 ± 13.1 #
% Collagen Area	6.56 ± 0.14	6.67 ± 0.22
Arterial stiffness and vessel morphometric analyses		
PWV (m/s)	3.92 ± 0.05	5.49 ± 0.35 #
**Aorta artery**		
% Collagen Area	14.6 ± 0.83	20.95 ± 1.16 #
Thickness (µm)	99.2 ± 3.95	103.4 ± 3.1
Thickness/lumen ratio	0.065 ± 0.002	0.068 ± 0.001
**Carotid artery**		
% Collagen Area	13.21 ± 1	20.11 ± 0.67 #
Thickness (µm)	48.74 ± 4.63	49.8 ± 2.1
Thickness/lumen ratio	0.067 ± 0.007	0.079 ± 0.004
**Femoral artery**		
% Collagen Area	15.75 ± 1.82	21.4 ± 0.92 #
Thickness (µm)	47.44 ± 2.65	47.8 ± 4.2
Thickness/lumen ratio	0.09 ± 0.006	0.08 ± 0.008

Body weight (BW); Systolic blood pressure (SBP); Diastolic blood pressure (DBP); Mean blood pressure (MBP); low frequency band (LF, nu); high frequency band (HF, nu); LF-HF ratio (LF/HF); Left ventricle systolic diameter (LVSD); Left ventricle diastolic diameter (LVDD); Left Ventricular Mass (LVM); Left Ventricular Mass Index (LVMI); Relative wall thickness (RWT); Posterior wall shortening velocity (PWSV); Myocardial performance index (TEI index); Ejection Fraction (LVEF); E/A ratio between early (E)-to-late (A) diastolic mitral inflow; Isovolumetric relaxation time (IVRT) and Pulse Wave Velocity (PWV) between sedentary animals: normotensive (Wistar, n=13) and hypertensive (SHR, n=11); Significance: # *vs* Wistar rats.

Table 5S. Morphometric data of Aorta, Carotid and Femoral Arteries in Wistar and all SHR Groups.

	Wistar	SC	SD	TC	TD
Aorta					
OD, µm	1,713 ± 36.1	1,717 ± 22.4	1,655.6 ± 29.5	1,688.8 ± 27.5	1,671.5 ± 19.6
ID, µm	1,514 ± 33.5	1,510.2 ± 19.8	1,448.4 ± 21.5	1,466.9 ± 23.3	1,463.9 ± 16.7
Wall thickness, µm	99.2 ± 3.9	103.4 ± 3.1	103.6 ± 6.2	110.9 ± 4.4	103.8 ± 4.3
Wall/lumen ratio	0.065 ± 0.002	0.068 ± 0.001	0.071 ± 0.003	0.075 ± 0.002	0.071 ± 0.003
Carotid					
OD, µm	709.3 ± 13.7	735.6 ± 12.9	714.1 ± 13.8	709.3 ±12.6	698.8 ± 17.3
ID, µm	625.8 ± 12.8	635.9 ± 13.5	616.2 ± 15.9	604.8 ± 16.1	604.3 ± 17.4
Wall thickness, µm	41.7 ± 4.6	49.8 ± 2.1	48.9 ± 1.9	52.2 ± 2.3	47.2 ± 2.2
Wall/lumen ratio	0.067 ± 0.007	0.079 ± 0.004	0.079 ± 0.004	0.087 ± 0.006	0.079 ± 0.004
Femoral					
OD, µm	530.5 ± 7.9	664.8 ± 18.3	619.9 ± 15.9	599.1 ± 14.6	611.5 ± 6.9
ID, µm	435.5 ± 9.5	569.1 ± 16.6	525.2 ± 17.6	503.7 ± 12.5	518.7 ± 7.3
Wall thickness, µm	47.4 ± 1.5	47.8 ± 4.2	47.21 ± 3.8	47.7 ± 2.9	46.3 ± 2.46
Wall/lumen ratio	0.10 ± 0.005	0.08 ± 0.008	0.09 ± 0.009	0.095 ± 0.005	0.09 ± 0.005

Outer Diameter (OD, µm); Inner Diameter (ID, µm); wall/Lumen ratio (wall thickness / ID) in Wistar (n = 13) and all SHR Groups: Sedentary control (SC, n = 8), Sedentary treated with DEX (SD, n=11), Trained control (TC, n = 12) and Trained treated with DEX (TD, n = 11).
